# Investigation of Interactions between Thrombin and Ten Phenolic Compounds by Affinity Capillary Electrophoresis and Molecular Docking

**DOI:** 10.1155/2018/4707609

**Published:** 2018-03-20

**Authors:** Qiao-Qiao Li, Yu-Xiu Yang, Jing-Wen Qv, Guang Hu, Yuan-Jia Hu, Zhi-Ning Xia, Feng-Qing Yang

**Affiliations:** ^1^School of Chemistry and Chemical Engineering, Chongqing University, Chongqing 401331, China; ^2^State Key Laboratory of Quality Research in Chinese Medicine, Institute of Chinese Medical Sciences, University of Macau, Macau, China; ^3^School of Pharmacy and Bioengineering, Chongqing University of Technology, Chongqing 400054, China

## Abstract

Thrombin plays a vital role in blood coagulation, which is a key process involved in thrombosis by promoting platelet aggregation and converting fibrinogen to form the fibrin clot. In the receptor concept, drugs produce their therapeutic effects via interactions with the targets. Therefore, investigation of interaction between thrombin and small molecules is important to find out the potential thrombin inhibitor. In this study, affinity capillary electrophoresis (ACE) and in silico molecular docking methods were developed to study the interaction between thrombin and ten phenolic compounds (*p*-hydroxybenzoic acid, protocatechuic acid, vanillic acid, gallic acid, catechin, epicatechin, dihydroquercetin, naringenin, apigenin, and baicalein). The ACE results showed that gallic acids and six flavonoid compounds had relative strong interactions with thrombin. In addition, the docking results indicated that all of optimal conformations of the six flavonoid compounds were positioned into the thrombin activity centre and had interaction with the HIS57 or SER195 which was the key residue to bind thrombin inhibitors such as argatroban. Herein, these six flavonoid compounds might have the potential of thrombin inhibition activity. In addition, the developed method in this study can be further applied to study the interactions of other molecules with thrombin.

## 1. Introduction

Thrombosis persists as a leading cause of death and incapacity worldwide [1]. The formation of thrombosis is a very complex pathological process involving the platelets and blood coagulation components. Thrombin plays a vital role in blood coagulation by promoting platelet aggregation and by converting fibrinogen to form the fibrin clot in the final step of the coagulation cascade [2]. Thrombin is composed of two polypeptide chains of 36 (A chain) and 259 (B chain) residues that are covalently linked through a disulfide bond, and the B chain carries the functional epitopes of the enzyme [3]. As same as all chymotrypsin-like serine proteases, thrombin has a conserved active centre located inside the molecule and contains amino acid residues of HIS57, ASP102, and SER195, which are called the catalytic triad [4]. Except for its active centre, thrombin possesses two exosites (1 and 2), positively charged domains located at opposite poles of the enzyme, among them, exosite 1 is utilized to dock on the substrates such as fibrinogen, and exosite 2 serves as the heparin-binding domain [5].

The current antithrombotic therapies include heparin (unfractionated heparin and low-molecular-weight heparins), fondaparinux, vitamin K antagonists, factor Xa inhibitors, and direct thrombin inhibitors [6]. Direct thrombin inhibitors (DTIs), such as argatroban, dabigatran, lepirudin, desirudin, and bivalirudin, which bind to thrombin and block its enzymatic activity, are widely and effectively used in the treatment of thromboembolic diseases; however, dabigatran is not orally available due to its high polarity [7]. Therefore, dabigatran etexilate as prodrug was developed to facilitate gastrointestinal absorption by adding an ethyl group at the carboxylic acid group and a hexyloxycarbonyl side chain at the amidine group [8]. Compared with heparins, DTIs do not require antithrombin as a cofactor and do not bind to plasma proteins; therefore, they produce a more predictable anticoagulant effect, and variability of patient response is low relative to other drug classes [9]. In reality, they still present limitations such as a narrow therapeutic window, and bleeding and anaphylaxis as side effects [10]. Therefore, alternative antithrombotic therapies are under extensive investigation, and many entities from natural products are being isolated and studied to counteract these side effects [11]. Liu et al. [12] evaluated a series of natural flavonoids as potential thrombin inhibitors by optimized method of thrombin time and found that myricetin and quercetin were the best thrombin inhibitors. Bijak et al. [4] showed that cyanidin, quercetin, and silybin changed thrombin proteolytic activity, while cyanidin and quercetin caused a strong response in the interaction with immobilized thrombin by BIAcore analyses. Thus, their results suggested that polyphenolic compounds might be potential structural bases and source to find and project nature-based, safe, orally bioavailable direct thrombin inhibitors.

In the receptor concept, drugs produce their therapeutic effects via interactions with the targets [13]. Therefore, evaluation of the interaction between thrombin and phenolic compounds is important for studying of drug action mechanism and drug discovery. Affinity capillary electrophoresis (ACE) is one of the predominant methods for interaction studies. Comparing with traditional methods such as equilibrium dialysis, ultrafiltration, ultracentrifugation, liquid chromatography, spectroscopic methods (UV-visible, fluorescence, infrared, nuclear magnetic resonance, optical rotatory dispersion, and circular dichroism), and isothermal titration calorimetry [14], ACE has lots of advantages including high separation efficiency, short analysis duration, low sample and reagent volumes, low purity requirement, ease of automation, and the ability to work under near-physiological conditions [14, 15]. At present, ACE had usually been applied in evaluating the noncovalent interaction between protein and ligands [15–17] or cell and ligands [13, 18]. In addition, in silico molecular docking had recently been presented as an assistant technology to obtain information about the interactions between thrombin and small molecules [19–21].

**Figure 1:**
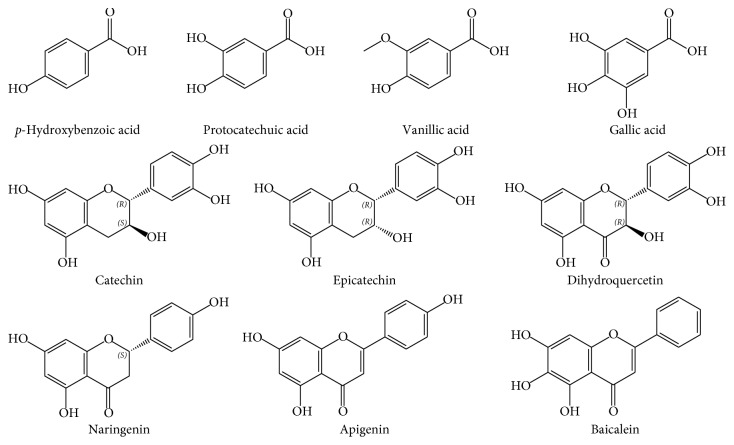
The chemical structures of ten investigated phenolic compounds.

The aim of this study was to investigate the interaction between thrombin and phenolic compounds including *p*-hydroxybenzoic acid, protocatechuic acid, vanillic acid, gallic acid, catechin, epicatechin, dihydroquercetin, naringenin, apigenin, and baicalein (the chemical structures of the investigated compounds are presented in Figure 1) by ACE and in silico molecular docking. Firstly, ACE was used to calculate the binding constants (*K*_b_) of the interactions between thrombin and phenolic compounds in experimental environment. Then, in silico molecular docking as assistant technology was also utilized to further explore the interactions between phenolic compounds and thrombin, including the binding sites and positioning of the ligand into the binding site. Finally, the interaction of compounds with enzyme activity centre, which might be potential thrombin inhibitors, could be obtained by the results of ACE and molecular docking.

## 2. Materials and Methods

### 2.1. Reagents

Bovine thrombin was obtained from Sigma-Aldrich (Shanghai) Trading Co., Ltd. (Shanghai, China). *p*-Hydroxybenzoic acid, vanillic acid, naringenin, apigenin, baicalein, catechin, and epicatechin were purchased from Chengdu Biopurify Phytochemicals Ltd. (Chengdu, China). Protocatechuic acid and dihydroquercetin were purchased from Push Bio-Technology Co., Ltd. (Chengdu, China). Gallic acid was the product of Aladdin Reagent Co., Ltd. (Shanghai, China). Tris(hydroxymethyl)aminomethane (Tris) was obtained from Sangon Biotech (Shanghai) Co., Ltd. (Shanghai, China). HCl and acetone were analytical grade reagents and purchased from Chengdu Kelong Chemical Reagent Factory (Chengdu, China).

### 2.2. Apparatus

The capillary electrophoresis experiments were performed on an Agilent 7100 3D CE system (Agilent Technologies, Palo Alto, CA, USA) equipped with a diode array detector and Agilent ChemStation software. The bare fused-silica capillary (Yongnian Ruifeng Chromatographic Device Co., Ltd., Hebei, China) was 75 *μ*m id and had a total length of 50 cm and an effective length of 41.8 cm. The water used for all the experiments was purified by water purification system (ATS-H20, Antesheng Environmental Protection Equipment Co., Ltd., Chongqing, China). Background electrolytes buffer and phenolic compound solutions were ultrasonicated in a KQ-100B ultrasonic cleaner (Kunshan Ultrasonic Instruments Co., Ltd., Kunshan, China). The pH of running buffer was measured by a FE28 pH meter (Mettler-Toledo Instruments, Shanghai, China).

### 2.3. Sample and Buffer Preparation

The running buffer containing 50 mol/L Tris was adjusted to pH 9.0 with 1 mol/L HCl. 10,000 U of thrombin was solved in 20 mL water, and the enzyme activity was 500 U/mL. The running buffers containing different concentrations of thrombin (0.4 U/mL, 0.8 U/mL, 1.2 U/mL, 1.6 U/mL, and 2.0 U/mL) were prepared by diluting the 500 U/mL thrombin stock solutions with running buffer. The samples of phenolic compounds (about 0.2 g/L) were prepared by dissolving each compound in buffer solution containing 5% (*v*/*v*) acetone as neutral EOF marker.

### 2.4. Electrophoresis Conditions

The voltage applied across the capillary was 20 kV. The sample was injected into the capillary under the pressure of 50 mbar for 5 s. The temperature of the capillary cartridge was set at 25°C. The conditioning between two successive runs was done according to the following protocol: 1 mol/L NaOH solution for 2 min, water for 2 min, and buffer solution for 2 min under 930 mbar.

### 2.5. Calculation of *K*_b_ by ACE

**Figure 2:**
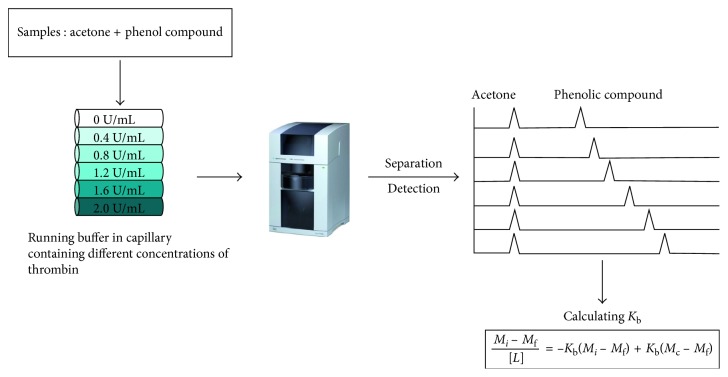
Schematic diagram of the steps of this study.

A schematic diagram of calculating *K*_b_ is shown in Figure 2. In ACE, *K*_b_ can be determined from the variation of mobility shifts of the sample at running buffer containing different concentrations of thrombin. The binding constant *K*_b_ could be calculated by the Scatchard equation [22] as follows:

(1) $$\begin{array}{c} \;\;\frac{\mu_{i}-\mu_{\text{f}}}{\left[L\right]}=-K_{\text{b}}\left(\mu_{i}-\mu_{\text{f}}\right)+K_{\text{b}}\left(\mu_{c}-\mu_{\text{f}}\right), \end{array}$$

where *K*_b_ is the binding constant, [*L*] is the equilibrium concentration of uncomplexed ligand, *μ*_f_ and *μ*_c_ are the electrophoretic mobility of free and complexed solute, and *μ*_*i*_ is the solute mobility measured at ligand concentration [*L*].

In this work, the mobility ratio *M* was inducted to eliminate the effects of the variation of running buffer, as defined in the following equation:

(2) $$\begin{array}{c} M=\frac{\mu_{\text{net}}}{\mu_{\text{eo}}}=\frac{\mu }{\mu_{\text{eo}}}+1=\frac{l_{\text{c}}l_{\text{d}}/\left(Vt\right)}{l_{\text{c}}l_{\text{d}}/\left(Vt_{\text{eo}}\right)}+1=\frac{t_{\text{eo}}}{t}+1, \end{array}$$

where *l*_*c*_ is the total length of the capillary, *l*_*d*_ is the effective length of the capillary, *μ*_net_ is the net mobility measured for the solute, *μ*_eo_ is the mobility due to EOF, *μ* is the inherent mobility of the solute, *t* is the measured analyte migration time, *t*_eo_ is the migration time of the neutral marker, and *V* is the operating voltage.

Analysis that can be performed using *M* is thus independent of the capillary length and voltage; the expression depends solely on the migration time of the phenolic compound relative to acetone. Equations (1) and (2) were integrated into (3). *K*_b_ could be obtained as follows:

(3) $$\begin{array}{c} \frac{M_{i}-M_{\text{f}}}{\left[L\right]}=-K_{\text{b}}\left(M_{i}-M_{\text{f}}\right)+K_{\text{b}}\left(M_{c}-M_{\text{f}}\right), \end{array}$$

where *M*_*i*_ is the solute mobility ratio measured at the ligand concentration [*L*] and *M*_f_ and *M*_c_ are the electrophoretic mobility ratio of free and complexed solute.

### 2.6. In Silico Molecular Docking

An in silico molecular docking study was performed to validate the binding potency of the phenolic compounds to thrombin by using AutoDock 4.2 program [23]. The molecular dockings were conducted by using the crystal structure of the thrombin-argatroban complex (PDB ID = 1DWC) at 1.53 Å resolution [24], where the ligand argatroban was deleted using UCSF Chimera. Besides, polar hydrogen atoms were added, and the crystal water was remained. The three-dimensional chemical structures of compounds were drawn by ChemOffice and minimized energy, with outputting in PDB format.

The cubic grid box was set to 60 × 60 × 60 points with a spacing of 0.375 Å. The catalytic site of the grid box was centralized using the following coordinates (*x* = 35.887; *y* = 19.178; *z* = 18.856). To find the best orientations and conformations of the ligands in the protein binding sites, the Lamarckian genetic algorithm was selected, with an initial population size of 150, a maximum number of evaluations of 2.5 × 10^6^ (medium), maximum number of generations of 27,000, gene mutation rate of 0.02, crossover rate of 0.8, and number of GA runs equal to 50 [25]. The interaction figures were generated, and the results of docking were recorded with binding potency and bonded residues. Additionally, the 2D interaction diagrams also were produced by Discovery Studio 4.5 to obtain specific interaction analysis including the functional groups, bonded residues, and interaction force.

## 3. Results and Discussion

### 3.1. ACE Analysis

Through preliminary investigation of thrombin concentration in running buffer, the six suitable concentrations were arranged as 0 U/mL, 0.4 U/mL, 0.8 U/mL, 1.2 U/mL, 1.6 U/mL, and 2.0 U/mL for ACE analyses. Considering about some flavonoid compounds were sparingly soluble in buffer at pH 7.4, and the activity of thrombin presents a maximum around pH 9.5 [26]. Therefore, the pH 9.0 of running buffer was used.

**Figure 3:**
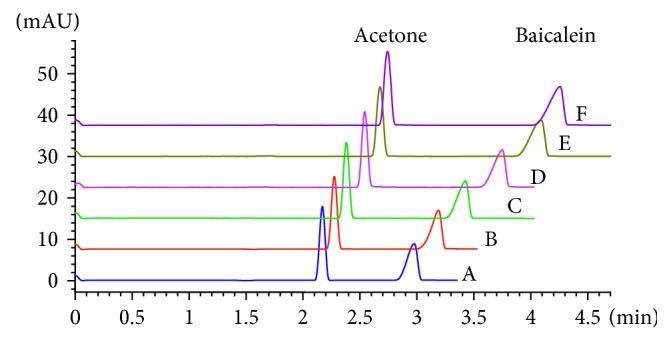
Electrophoregrams of baicalein and acetone in running buffers containing different concentrations of thrombin. Thrombin concentration in running buffer: 0 U/mL (a), 0.4 U/mL (b), 0.8 U/mL (c), 1.2 U/mL (d), 1.6 U/mL (e), and 2.0 U/mL (f).

It was attempted to calculate the *K*_b_ value of argatroban and thrombin. However, the migration time of argatroban was same as argatroban-thrombin complex which did not meet the basic requirement of the ACE method [14]. So the *K*_b_ value of interaction between argatroban and thrombin could not be obtained in this study. Baicalein as sample containing 5% (*v*/*v*) acetone was analyzed in running buffer at six concentrations. As shown in Figure 3, the migration of acetone delayed due to the effect of the variation of running buffer. But the introduced mobility ratio eliminated the error. Then, the binding constant was calculated by the variation of mobility shifts of baicalein at running buffer containing different concentrations of thrombin. The *K*_b_ values of other compounds were calculated by the same procedure (electrophoregrams of other compounds were provided in Supplementary Figure S1). The migration time of *p*-hydroxybenzoic acid, vanillic acid, and protocatechuic acid delayed, and mobility ratio became smaller with increasing the thrombin concentration in running buffer which indicated that thrombin had interaction with them. However, there was no nonlinear correlation between (*M*_f_ − *M*_*i*_)/[*L*] and (*M*_f_ − *M*_*i*_). According to the previous study [26], the binding modes of small molecules and biomacromolecules include site-specific and nonspecific binding. Furthermore, the *K*_b_ value could only be calculated by the Scatchard equation when the site-specific binding mode was dominant. Therefore, the reason of *K*_b_ value of interaction of thrombin and *p*-hydroxybenzoic acid, vanillic acid, and protocatechuic acid could not be obtained in this study might be that the site-specific binding force was weak. The other phenolic compounds had relative stronger affinity with thrombin, and the *K*_b_ values are shown in Table 1.

In general, the affinities of thrombin binding with flavonoids were stronger than phenolic acids according to the *K*_b_ values obtained by ACE. Gallic acid possessed more OH group than other three phenolic acid compounds (*p*-hydroxybenzoic acid, vanillic acid, and protocatechuic acid), which may relate to its stronger affinity with thrombin. Compared with the *K*_b_ values of other flavonoid compounds, baicalein had the strongest affinity with thrombin, which is possibly contributed to the existence of three OH groups (5th, 6th, and 7th positions) at A-ring. Liu et al. [12] demonstrated that more OH groups in the A-ring will increase thrombin inhibition activity. Previous reported results also indicated that hydroxyl groups at C-3′ and C-4′ positions in the B-ring and hydroxyl group at C-3 position in the C-ring played key roles in the thrombin inhibitory activity [27]. And the present results of ACE showed that naringenin had the lowest affinity with thrombin due to lack of OH groups. According to the calculated *K*_b_ values of naringenin and apigenin, the presence of C2=C3 was also important for the affinity with thrombin. Additionally, spatial structure difference could result in different binding strength to thrombin such as catechin and epicatechin.

### 3.2. Molecular Docking Results

The essence of molecular docking was a recognition process of two or more molecules, involving the space and energy matching between them. The major tasks of the docking procedure were characterization of the binding site, positioning of the ligand into the binding site, and evaluating the strength of interaction for a specific ligand-receptor complex [28]. The semiflexible docking was applied in this study. In the docking, the thrombin conformation kept constant, but the compound conformation could change slightly to get the most stable phenolic compound-thrombin complex also called optimal conformation.

**Figure 4:**
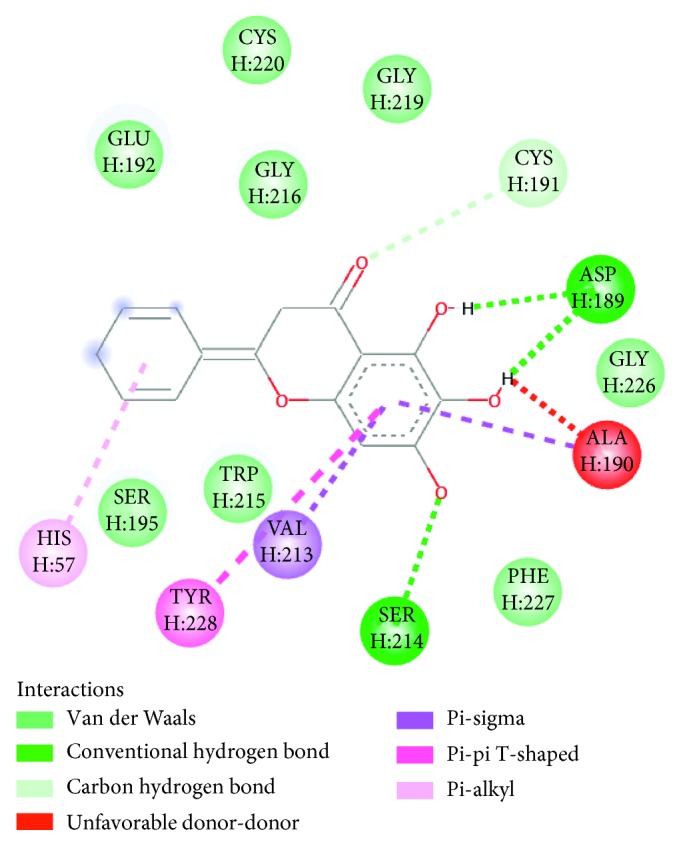
The 2D interaction diagram of baicalein with residues of thrombin.

Clustering the docking conformations of thrombin-phenolic acid complexes, the result showed that their optimal conformation was not present in the activity centre, meaning that the four phenolic acid compounds could bind with other place of the enzyme. Therefore, this study only discussed the docking results of thrombin and flavonoid compounds. The 2D interaction diagram of baicalein with residues of thrombin can be observed in Figure 4. Three OH groups (5th, 6th, and 7th positions) at A-ring of baicalein have interaction with ASP189 and SER214 of thrombin by hydrogen bonds which are marked by deep green dotted lines on the 2D interaction diagram. The 2D interaction diagrams of other compounds with residues of thrombin are shown in Supplementary Figure S2, and the docking results and residue interactions are demonstrated in Table 2.

**Figure 5:**
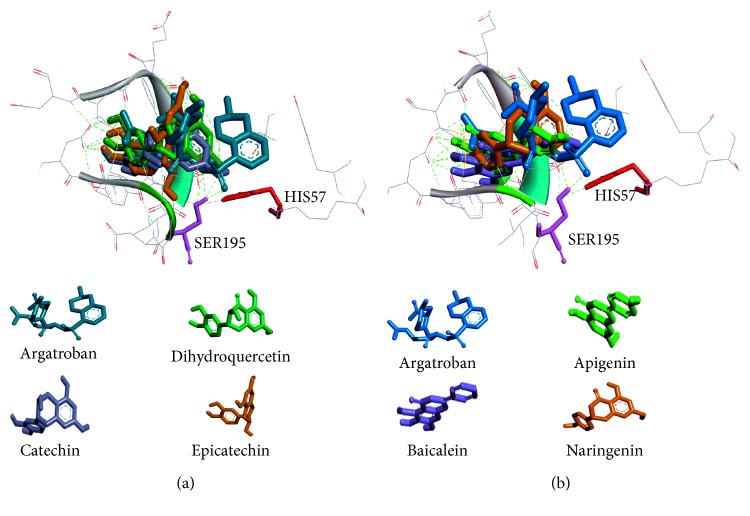
Comparison of 3D structures of six flavonoids docked with the thrombin catalytic site. (a) The 3D structures of argatroban, dihydroquercetin, catechin, and epicatechin; (b) the 3D structures of argatroban, apigenin, baicalein, and naringenin.

It was recognized that the region with binding energy below −5.0 kcal/mol could be regarded as the “Potential Targets” [23]. The docking results showed that the binding energies of six flavonoid compounds were similar and below −7.0 kcal/mol. A possible explanation is that their highly similar structures lead to the approximate docking results. Therefore, all of six compounds had relative strong affinity with thrombin. In addition, the binding positions of six flavonoid compounds were alike with argatroban as shown in Figures 5(a) and 5(b). It is worth mentioning that all of them located in the activity centre and had interaction with the residue HIS57 or SER195, which indicated that these compounds likely had thrombin inhibitory activity.

## 4. Conclusion

In this work, quick and simple methods (ACE and in silico molecular docking) were developed to investigate the interactions between thrombin and ten phenolic compounds. As compared with conventional methods for interaction study, combination of ACE and molecular docking could greatly decrease the analyses time and sample consumption, and the mechanisms of drug effect could be explained by specific interaction with residues of proteins. Furthermore, the results of interaction study in actual (experimental) and simulation environment are mutually complementary or verified.

The results of present study indicated that the interactions between thrombin and phenolic acids were weak except for gallic acids. On the other hand, in docking results, phenolic acids could only interact with residues other than activity centre due to lack of space matching. So these phenolic acids (except gallic acid) possibly have not thrombin inhibition effects. The calculated *K*_b_ values by ACE in actual environment indicated that gallic acid and six flavonoid compounds had relative strong affinity with thrombin, and the affinity was positively correlated with the number of OH groups in A-ring specially. Furthermore, OH groups at C-3′ and C-4′ position in the B-ring and OH group at C-3 position in the C-ring, and the presence of C2=C3 also played key roles for the interaction with thrombin. The docking results showed that all of optimal conformations of the investigated flavonoid compounds were positioned into the thrombin activity centre and had interactions with HIS57 or SER195, which indicated that these flavonoids possibly had the thrombin inhibition activity. In a word, combination of ACE and molecular docking is an effective approach for studying the interaction between thrombin and small molecules, and characterizing of the binding site, which is an important process for drug discovery.

## Figures and Tables

**Table 1:** Interactions of ten investigated compounds with thrombin evaluated by ACE.

Compounds	Molecule weight	p*K*a	Detection wavelength (nm)	Regression equation of (*M*_f_ − *M*_*i*_)/[*L*] and (M_f_−M_*i*_)	Binding constant (mL/U)
*p*-Hydroxybenzoic acid	138.12	4.57 ± 0.10	250	—	—
Protocatechuic acid	154.12	4.45 ± 0.10	250	—	—
Vanillic acid	168.15	4.45 ± 0.10	250	—	—
Gallic acid	170.12	4.33 ± 0.10	250	*y* = −0.184*x* + 0.105 (*R* ^2^ = 0.95)	0.184
Naringenin	272.25	7.52 ± 0.40	280	*y* = −0.238*x* + 0.117 (*R* ^2^ = 0.99)	0.238
Apigenin	270.24	6.53 ± 0.40	280	*y* = −0.302*x* + 0.147 (*R* ^2^ = 0.99)	0.302
Baicalein	270.24	6.31 ± 0.40	280	*y* = −0.508*x* + 0.093 (*R* ^2^ = 0.94)	0.508
Catechin	290.27	9.54 ± 0.10	280	*y* = −0.353*x* + 0.080 (*R* ^2^ = 0.98)	0.353
Epicatechin	290.27	9.54 ± 0.10	280	*y* = −0.297*x* + 0.043 (*R* ^2^ = 0.99)	0.297
Dihydroquercetin	304.25	7.39 ± 0.60	250	*y* = −0.389*x* + 0.075 (*R* ^2^ = 0.99)	0.389

**Table 2:** The docking results and residue interactions of the complexes of argatroban and six flavonoids with thrombin.

Compounds	Molecule weight	NC	NP	BE (kcal/mol)	H-bond	EI	VDW
Argatroban	508.63	26	7	−8.93	**HIS57**, ASP189, ALA190, **SER195**, GLY219	TYR60A, TRP60D, LEU99, TRP215	CYS60F, CYS191, GLU192, GLY193, VAL213, SER214, GLY216, GLU217, CYS220, GLY226, PHE227
Baicalein	270.24	1	50	−7.31	ASP189, CYS191, SER214	**HIS57**, ALA190, VAL213, TYR228	GLU192, **SER195**, TRP215, GLY216, GLY219, CYS220, GLY226, PHE227
Apigenin	270.24	1	50	−7.39	ASP189, GLU192, GLY226, PHE227	ALA190, VAL213, TYR228	CYS191, **SER195**, SER214, TRP215, GLY216, GLY219, CYS220
Naringenin	272.25	1	50	−7.80	ASP189, SER214, GLY216, GLY219	ALA190, CYS191, CYS220	**HIS57**, LEU99, GLU192, **SER195**, VAL213, TRP215, GLU217, GLY226, PHE227, TRY228
Dihydroquercetin	304.25	4	42	−7.67	ASP189, SER214, GLY216, GLY219	ALA190, CYS220	**HIS57**, LEU99, CYS191, GLU192, **SER195**, VAL213, TRP215, GLU217, ASP221, GLY226, PHE227, TYR228
Catechin	290.27	4	27	−7.59	ASP189, **SER195**, SER214, GLY216, CYS220, TYR228	ALA190, VAL213, TYR228	**HIS57**, CYS191, GLU192, TRP215, GLY219, GLY226, PHE227
Epicatechin	290.27	3	45	−8.09	ASP189, GLU192, **SER195**, SER214, GLY216, GLY219, CYS220, TYR228	ALA190, VAL213, TRP215	**HIS57**, CYS191, ASP194, GLY226, PHE227

NC: number of clusters; NP: number of poses on the lowest energy cluster; BE: binding energy; H-bond: hydrogen bond; EI: electrostatic interactions; VDW: van der Waals.

## Supplementary Materials

Supplementary Materials Figure S1: electrophoregrams of phenolic compounds and acetone in running buffers containing different concentrations of thrombin. Thrombin concentration in running buffer: 0 U/mL (A), 0.4 U/mL (B), 0.8 U/mL (C), 1.2 U/mL (D), 1.6 U/mL (E), and 2.0 U/mL (F). (a) epicatechin; (b) catechin; (c) dihydroquercetin; (d) naringenin; (e) apigenin; (f) gallic acid; (g) *p*-hydroxybenzoic acid; (h) protocatechuic acid; and (i) vanillic acid. Figure S2: the 2D interaction diagrams of investigated compounds with residues of thrombin. (a) epicatechin; (b) catechin; (c) dihydroquercetin; (d) naringenin; (e) apigenin; and (f) argatroban.
