# Supplementary material for: Investigation of Interactions between Thrombin and Ten Phenolic Compounds by Affinity Capillary Electrophoresis and Molecular Docking
Source: J Anal Methods Chem. 2018 Mar 20;2018:4707609. doi: 10.1155/2018/4707609 (PMC5884136; doi:10.1155/2018/4707609)
Supplement: Supplementary Materials — Figure S1: electrophoregrams of phenolic compounds and acetone in running buffers containing different concentrations of thrombin. Thrombin concentration in running buffer: 0 U/mL (A), 0.4 U/mL (B), 0.8 U/mL (C), 1.2 U/mL (D), 1.6 U/mL (E), and 2.0 U/mL (F). (a) epicatechin; (b) catechin; (c) dihydroquercetin; (d) naringenin; (e) apigenin; (f) gallic acid; (g) p-hydroxybenzoic acid; (h) protocatechuic acid; and (i) vanillic acid. Figure S2: the 2D interaction diagrams of investigated compounds with residues of thrombin. (a) epicatechin; (b) catechin; (c) dihydroquercetin; (d) naringenin; (e) apigenin; and (f) argatroban. [file 4707609.f1.pdf]

Supporting Information

Investigation of Interactions between Thrombin and Ten Phenolic Compounds by Affinity Capillary Electrophoresis and Molecular Docking

Qiao-Qiao Li[1], Yu-Xiu Yang[1], Jing-Wen Qv[2], Guang Hu[3], Yuan-Jia Hu[2]*, Zhi-Ning Xia[1], Feng-Qing Yang[1]*

[1] School of Chemistry and Chemical Engineering, Chongqing University, Chongqing 401331, PR China

[2] State Key Laboratory of Quality Research in Chinese Medicine, Institute of Chinese Medical Sciences, University of Macau, Macao, PR China

[3] School of Pharmacy and Bioengineering, Chongqing University of Technology, Chongqing 400054, China

*Addressed for correspondence:

Prof. Dr. Feng-Qing Yang, School of Chemistry and Chemical Engineering, Chongqing University, Chongqing 401331, China. E-mail: fengqingyang@cqu.edu.cn, ysfhqx@hotmail.com

Dr. Yuan-Jia Hu, State Key Laboratory of Quality Research in Chinese Medicine, Institute of Chinese Medical Sciences, University of Macau, Macao SAR, China. E-mail address: yuanjiahu@umac.mo

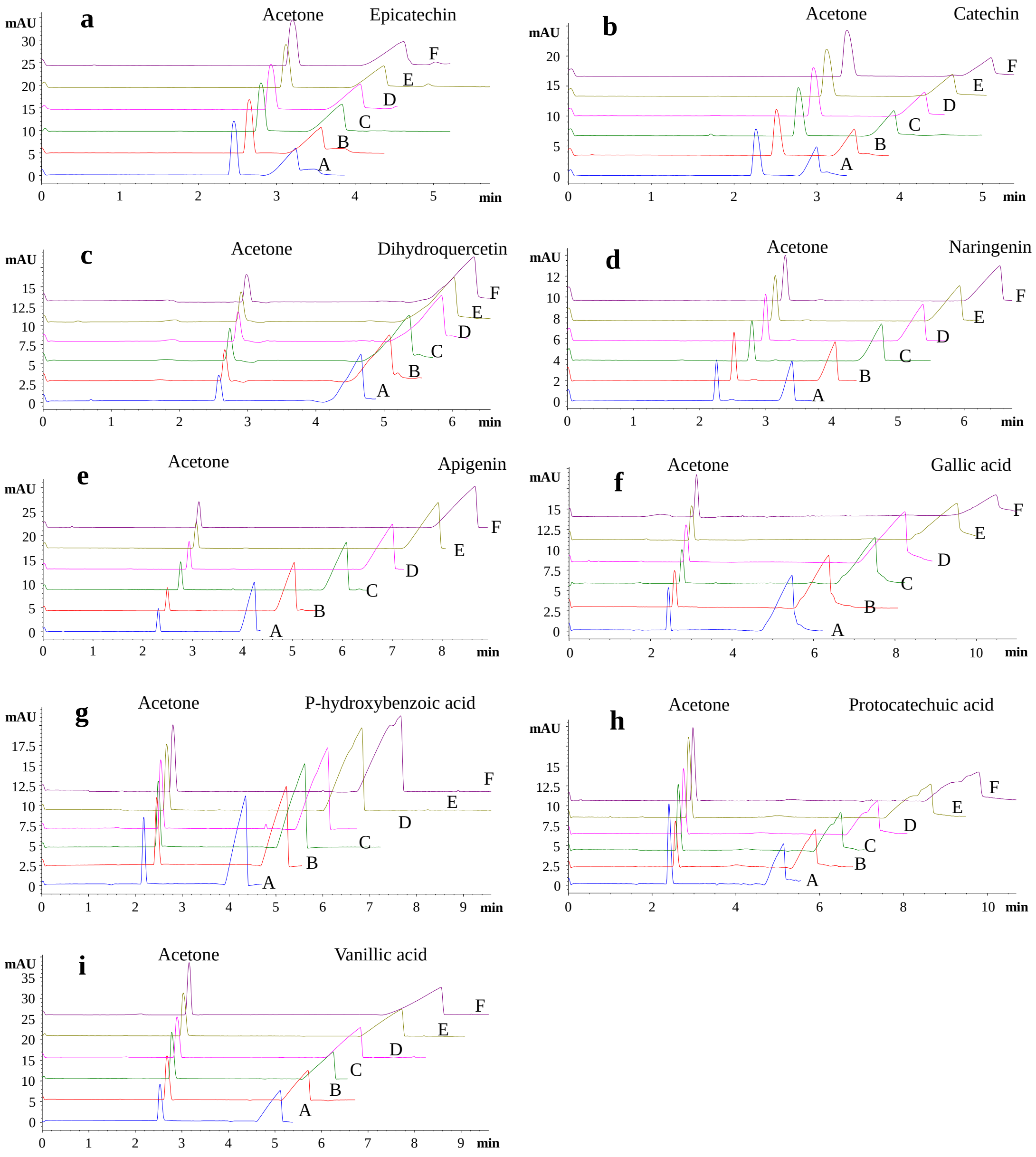

Figure S1 Electrophoregrams of phenolic compounds and acetone in running buffers containing different concentrations of thrombin. Thrombin concentration in running buffer: 0 U/ mL (A), 0.4 U/ mL (B), 0.8 U/ mL (C), 1.2 U/ mL (D), 1.6 U/ mL (E), 2.0 U/ mL (F). a: epicatechin; b: catechin; c: dihydroquercetin; d: naringenin; e: apigenin; f: gallic acid; g: p-hydroxybenzoic acid; h: protocatechuic acid; i: vanillic acid.

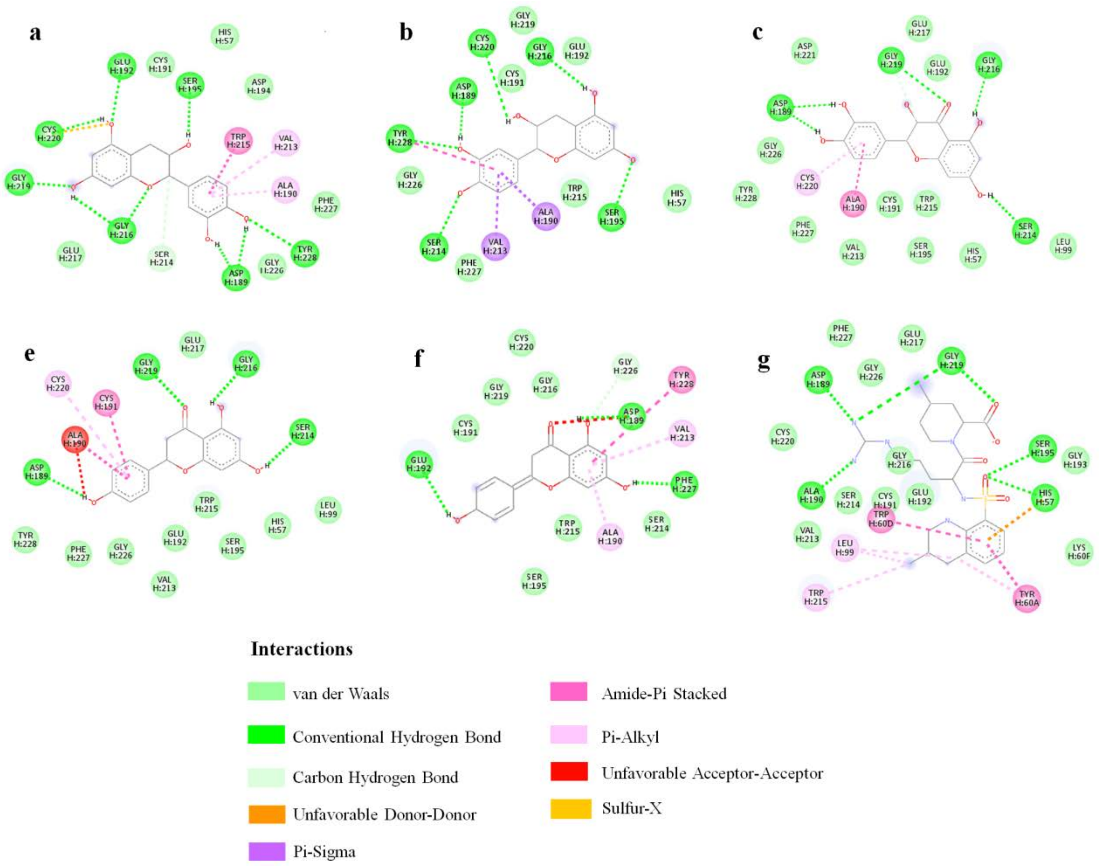

Figure S2 The 2D interaction diagrams of investigated compounds with residues of thrombin. a: epicatechin; b: catechin; c: dihydroquercetin; d: naringenin; e: apigenin; f: argatroban.
